# Dietary carbohydrate intake in patients with type 2 diabetes mellitus and diabetes control: a cross-sectional study

**DOI:** 10.29219/fnr.v64.4751

**Published:** 2020-11-16

**Authors:** Muneera Q Al-Mssallem, Ali A Al-Qarni, Mohammed Al-Jamaan

**Affiliations:** 1 Department of Food Sciences and Nutrition, Faculty of Agricultural and Food Sciences, King Faisal University, Al-Ahsa, Saudi Arabia; 2 King Abdullah International Medical Research Centre, Eastern Region, Ministry of National Guard Health Affairs, Al-Ahsa, Saudi Arabia; 3 Primary Health Care, King Abdullah Military Housing, Ministry of National Guard Health Affairs, Eastern Region, Al-Ahsa, Saudi Arabia

**Keywords:** available carbohydrate, blood glucose, HbA_1c_, macronutrients, type 2 diabetes

## Abstract

**Background:**

Food intake has important implications for patients with type 2 diabetes.

**Objective:**

Similarly, in other crop species, this observational study aimed to assess dietary carbohydrate (CHO) and non-starch polysaccharide (NSP) intake and examine their association with glycemic control among Saudi patients with type 2 diabetes mellitus (T2DM).

**Design:**

We investigated dietary intake in 404 patients (207 males and 197 females) with T2DM between November 2018 and March 2019. Dietary intake was assessed by face-to-face interviews using a validated dietary questionnaire.

**Results:**

The results revealed that dietary CHO intake (67% of energy) exceeded the recommended daily intake, and white rice (Basmati rice) was the major contributor to CHO intake. However, the dietary NSP intake was lower than recommended, and it was negatively associated with HbA_1c_ levels.

**Conclusion:**

This cross-sectional study showed that dietary CHO intake was high among Saudi patients with type 2 diabetes, and that their daily intake of NSPs was correlated with a lower level of HbA_1c_. Dietary advice should be given for patients with diabetes to reduce their intake of starchy food such as rice.

## Popular scientific summary

- The dietary intake of carbohydrate among patients with type 2 diabetes exceeded the recommendation
- The major contribution to carbohydrate intake was white rice
- The dietary intake of non-starch polysaccharides was below the recommendation
- The higher intake of non-starch polysaccharides was associated with lower HbA1c level

Lifestyle and, in particular, diet play a crucial role in the burden of chronic conditions such as type 2 diabetes mellitus (T2DM) (1). T2DM is a chronic disease that is characterized by aberrations in glucose and insulin metabolism, and aberrations are postulated to increase the risk of developing cardiovascular and other vascular complications (2). Individuals with T2DM have been the center of much research involving dietary manipulation to reduce morbidity and mortality (3). Historically, individuals with T2DM have been advised to restrict the amount of carbohydrates (CHOs) they eat and spread the total throughout the day to reduce both glucose and insulin aberrant levels and reduce complications (4).

In fact, dietary CHOs are fundamental macronutrients in terms of their capacity to influence blood glucose and insulin levels (5). However, the chemical nature of CHOs is not a reliable indicator of their physiological effects. Therefore, the term ‘glycaemic index (GI)’ was introduced, and it is an approach for classifying foods according to their physiological impact on blood glucose levels (6). The GI is a useful parameter that can be used to aid our understanding of the metabolic impact of different types of CHO-containing foods (7). In practice, the GI value is not directly related to the actual amount of CHOs content present in the food as eaten. The GI compares the blood glucose response to the specific amount of available CHOs (usually 50 g) present in a food (6). The available CHOs are usually computed by excluding non-starch polysaccharides (NSPs) from the total CHOs of a food (8, 9).

Several attempts to identify the optimal mix of macronutrients in meal plans for patients with diabetes have been addressed. However, there is no ideal distribution of macronutrient proportions for patients with diabetes (10). The Dietary Guidelines for patients with diabetes state that the adequate intake values of CHOs, fat, and protein consumption are 45–60%, 20–35%, and 15–20% of total energy intake, respectively (11, 12). NSPs are included in total CHOs, for which their recommended daily intake should be 25–38 g/day (13).

Indeed, dietary CHOs can be digested at differing rates and to different extents in the human small intestine (14). A wide range of effects on the glycemic response have been observed after the consumption of various proportions of CHOs in the form of monosaccharides, disaccharides, polysaccharides, and NSPs. In practice, a meal with sufficient dietary NSPs is likely to be absorbed more slowly and thus delay the elevation of blood glucose and insulin levels in patients with T2DM (15). The NSPs in foods consist of soluble and insoluble NSPs. Both fractions of NSPs play an important role in delaying gastric emptying and lowering the digestion and absorption of CHOs. It is evident that high NSP foods have a protective role in the development of diabetes (16). Several epidemiological studies have shown a significant association between dietary NSPs and decreased risk of diabetes (5, 17–19). Saudi Arabia is facing an epidemic of diabetes mellitus, where the prevalence of it is increasing at a considerable rate at approximately 25% among Saudi adults (20). This could be partially due to rapid changes in lifestyle and habitual eating patterns, in particular, the quantity and quality of dietary CHOs (21). A modification in the type and amount of dietary CHOs intake is known to alter the impact of dietary CHOs on the plasma glucose and insulin profiles, and this presents a useful strategy to alleviate some of the problems associated with T2DM. It is important to investigate the main source of dietary CHOs intake in relation to glycemic control among Saudi patients with diabetes. Therefore, this observational study aimed to assess the dietary available CHOs and NSP intake and examine their relationship to glycemic control among Saudi patients with T2DM.

## Patients and method

A total of 404 (207 males and 197 females) Saudi patients with type 2 diabetes aged 55.27 ± 9.66 years were recruited from the primary health care center, National Guard Health Affairs, Eastern Province, Al-Ahsa, Saudi Arabia. The sample size was calculated to provide an estimation of proportion with an 80% power, 95% confidence of interval (CI), and 5% margin of error. The study was conducted between November 2018 and March 2019. Pregnant women and patients with chronic kidney and liver diseases or on medications that affect diabetic control, e.g. glucocorticoids, were excluded from this study.

Dietary macronutrient intake and NSP intake were assessed by a validated food frequency questionnaire. The questionnaire consisted of 98 food items, and it has been reviewed by an academic nutritionist, an epidemiologist, and an endocrinology consultant. Additionally, a pilot study was conducted on 20 patients with type 2 diabetes, and a modification has been applied on the revised questionnaire accordingly (22). Patients have been interviewed face to face by a well-trained dietitian. The frequency of food intakes was reported using a four-point scale (daily, weekly, monthly, and never) beside reporting the amount of each food items. The portion size was designated using food models and other demonstrations. All data of each food intakes were converted to times per day, and then the portion size of each food items per day was calculated. The daily intakes of energy, macronutrients, and NSPs were estimated for each food items using the food composition tables for Arab Gulf citizens (23).

Systolic and diastolic blood pressures (Philips, Suresigns VS2 vital signs monitor, Andover, MA, USA), height, and weight (Adam, MDW 250L scale, Brooklyn, USA) were taken at the first visit to the diabetic clinic. Body mass index (BMI) for patients was calculated. Fasting blood glucose (FBG) and random blood glucose (RBG) levels, glycated hemoglobin HbA_1c_, triglycerides (TG), high-density lipoprotein (HDL), low-density lipoprotein (LDL), and total cholesterol (TC) were retrieved from patients’ electronic medical records that were available at the time of the interview.

This cross-sectional study was approved by the Institutional Research Board (IRB, Ref. No. IRBC/0666/19), Ministry of National Guard Health Affairs. An informed written consent was obtained from each patient.

### Statistical analysis

Data were analyzed using Statistical Package for Social Sciences (SPSS software, Version 21.0). Descriptive statistics were addressed through calculation of the mean and standard deviation of laboratory test results, food items, and total nutrient consumption among patients with diabetes with proportions of major nutrients for every food item. This study has used inferential statistical tests with the consideration of 5% two-tailed significance level (*P* value < 0.05). In addition, we measured nutrient consumption among patients with type 2 diabetes based on the recommended consumption using one sample *t*-test. The correlation between food items and laboratory test measures and between laboratory test measures and nutrient consumption among patients with diabetes was assessed using the Pearson correlation test.

## Results

### General characteristics of patients

Table 1 demonstrates the characteristics of the patients with type 2 diabetes. The mean diastolic and systolic blood pressures are within the normal range (<90 and <140 mmHg, respectively). The mean BMI was 33.79 ± 6.08 kg/m^2^, indicating that patients suffer from obesity. In addition, the means of HbA_1c_, FBG, and RBG were higher in comparison with the normal range.

**Table 1:** General characteristics of the patients with type 2 diabetes mellitus (*n* = 404, 207 males [51%] and 197 females [49%])

Measurement	Mean ± SD	Reference value*
Age (year)	55.27 ± 9.66	–
Weight (kg)	87.30 ± 16.53	–
Height (m)	1.60 ± 0.09	–
BMI (kg/m^2^)	33.79 ± 6.08	18.5-24.9
DBP (mmHg)	72.57 ± 10.47	70-90
SBP (mmHg)	138.66 ± 18.22	120-140
HbA_1c_ (%)	8.23 ± 1.41	4.40-6.40
FBG (mmol/L)	9.72 ± 3.52	3.90-5.60
RBG (mmol/L)	11.20 ± 4.21	2.90-7.80
TC (mmol/L)	4.40 ± 0.91	≤5.18
HDL (mmol/L)	1.04 ± 0.24	≥1.55
LDL (mmol/L)	2.70 ± 0.80	≤2.60
TG (mmol/L)	1.66 ± 0.93	≤1.70

BMI, body mass index; DBP, diastolic blood pressure; SBP, systolic blood pressure; HbA_1c_, glycated hemoglobin; FBG, fasting blood glucose; RBG, random blood glucose; TC, total cholesterol; HDL, high-density lipoprotein; LDL, low-density lipoprotein; TG, triglycerides.

* Sources: ADA (2020) and Evert et al. (2014).

### Food intake

**Fig. 1:**
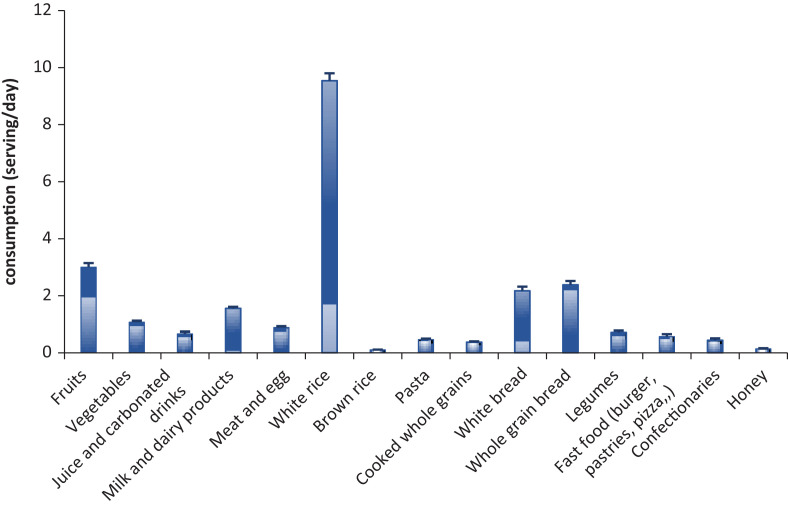
The daily intake of different foods (serving/day) among patients with type 2 diabetes (*n* = 404).

In this study, the daily intake of grains and grain-based products reached 15.6 servings/day, which exceeded the maximum recommended daily intake of this group. The highest portion came from Basmati rice, which accounted for 9.5 servings/day (Fig. 1). Thus, Basmati rice was the main source of CHOs in patients’ daily food intake, accounting for 43.1% of the total CHOs (Table 2). Breads (white and whole grains) were second in terms of their daily consumption, which reached approximately 4.5 servings/day. Breads contributed to CHOs with 20.6% of total CHOs (Table 2).

**Table 2:** The daily intake of different foods and their total energy, CHOs, available CHOs, protein, fat, and NSPs among patients with type 2 diabetes (*n* = 404)

Food intake	Total CHO (%)	Available CHO (%)	Protein (%)	Fat (%)	NSPs (%)	Energy (%)
Fruits (fresh, canned, dried)	13.6	10.9	–	–	41.4	9.1
Vegetables (fresh, cooked)	1.6	0.7	–	–	11.1	1.4
Juice and carbonated drinks	3	3.3	–	–	–	2
Milk and dairy products	5.6	6.2	19	24.1	–	11.3
Red and white meat plus egg	0	0	9.5	12.2	–	3.8
White rice (Basmati rice)	43.1	46.2	33.3	40.2	10.5	40.8
Brown rice (Hassawi rice)	0.5	0.4	0.4	0.4	0.8	0.4
Pasta	2.1	2.1	2.1	1	1.9	1.9
Cooked whole grains (Hareecee, Jeraish, Marqooq)	1.7	1.6	1.4	1.6	3.3	1.6
White bread	9.8	10.4	6.6	2.3	3.7	7.7
Whole grain bread	10.8	10.2	7.2	2.5	16.5	8.4
Legumes	3.2	2.6	16.6	7.5	9.8	5.9
Fast food (burger, pastries, pizza)	2.6	2.7	2.6	3.6	1	2.9
Confectionaries	2	2.2	1.3	4.6	0	2.5
Honey	0.4	0.5	–	–	–	0.3
Total	100	100	100	100	100	100

CHO, carbohydrate; NSPs, non-starch polysaccharides.

On the other hand, the daily intake of the vegetables, milk, and meat groups was 1.08, 1.56, and 0.89 servings/day, respectively. This intake of these both groups did not meet the recommended daily intake of these groups.

It is obvious that the highest contribution to the NSPs comes from fruits with a percentage of 41.37%, followed by whole grain bread and vegetables with values of 16.44% and 11.13%, respectively (Table 2).

The contribution of CHOs to the total daily energy reached 67%, which significantly exceeded the recommended dietary allowance (*P* < 0.001). However, protein, fat, and NSPs (Table 3) were significantly lower than recommended (*P* < 0.001).

**Table 3:** The levels of daily intake of macronutrients and NSPs in comparison to the recommended dietary intake* of these nutrients

Nutrients	Mean	P
CHO% of energy	67.09 ± 4.26	<0.001
Protein% of energy	13.15 ± 1.94	<0.001
Fat% of energy	21.41 ± 2.73	<0.001
NSPs g/1000 calorie	14.87 ± 5.00	<0.001

* Sources: ADA (2019) and Evert et al. (2019).

**Fig. 2:**
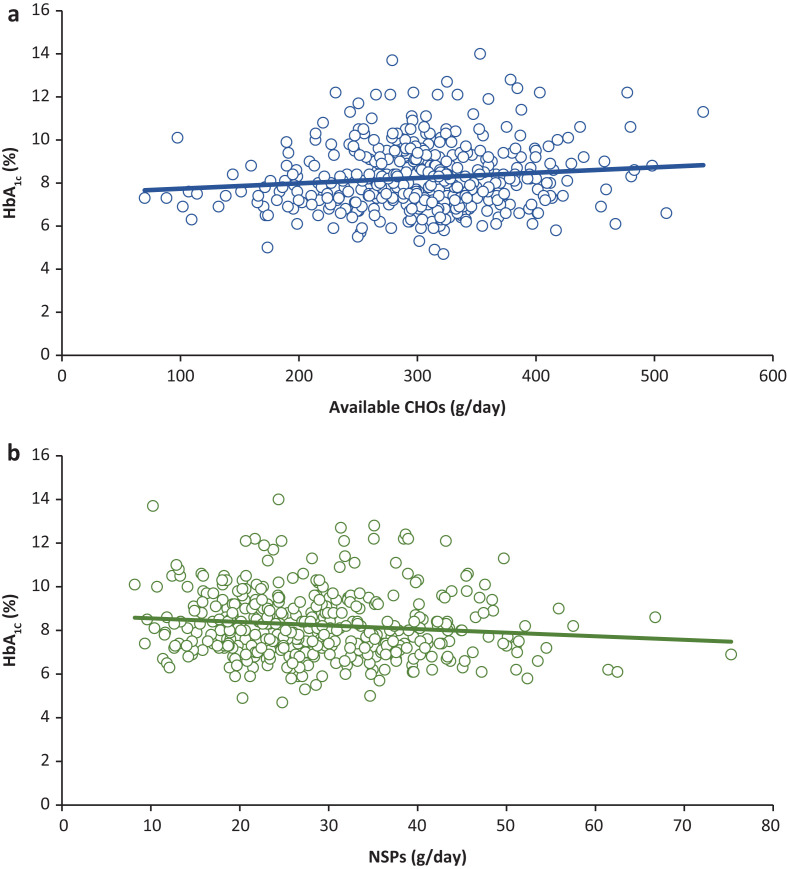
The positive (A) and negative (B) correlation of HbA_1c_ level with the daily consumed available CHOs and NSPs among patients with type 2 diabetes (*r* = 0.11, *P* = 0.01 and *r* = -0.11, *P* = 0.02, respectively).

Additionally, our results revealed that there was a weak positive correlation between HbA_1c_ and the daily intake of available CHOs (*r* = 0.11, *P* < 0.05, Fig. 2A). On the other hand, dietary NSP intake was significantly associated with a lower level of HbA_1c_ (*r* = -0.11, *P* < 0.05, Fig. 2B).

## Discussion

Modification of the type of CHOs content in the diet is behind most health problems. The main purpose of this study was to investigate the main source of CHOs and NSPs and examine their association with HbA_1c_ among patients with type 2 diabetes. A positive association was observed between dietary intake of CHOs and HbA_1c_, whereas NSPs intake was negatively correlated with HbA_1c_.

Our results have shown that patients had a high intake of CHOs. It seems that their intake of CHOs has exceeded the daily recommended intake of CHOs for patients with diabetes by approximately 21%. However, the daily intake of both protein and fat fell in the normal range. In fact, there is no perfect mix of macronutrients for patients with diabetes (24). However, some guidelines recommend 45–60% of total energy from CHOs, 10–20% as protein, and less than 35% from fat (11, 15).

In this study, patients’ daily consumption of vegetables, milk, and meat groups was below the recommended daily intake of these groups. However, the intake of grain and grain products exceeded the recommendation. The highest food intake in the patients’ diets was Basmati rice. In this study, Basmati rice was the main contributor to the CHOs and accounted for approximately 43% of the total CHOs. In Saudi Arabia, rice is usually cooked with vegetables and meat in a main dish, the so-called Kabsa (25). Kabsa is served mainly at lunch and is traditionally consumed at a high rate among the Saudi population. Fortunately, the estimated GI of Kabsa with Basmati rice falls in the low level, which ranges from 52 to 55. On the other hand, the calculated Glycaemic load (GL) of Basmati rice Kabsa ranged from 15 to 16, which is considered as medium value (26). This GL value would be as high as nine times for patients’ Basmati rice Kabsa intake in this study because of their high consumption. A negative correlation between BMI and the consumption of Basmati rice Kabsa was reported among Saudi population (26). However, this association has not been observed in this study. In fact, there was a positive association between the available CHO intake among patients in this study and the HbA1c level. As the Basmati rice was the main source of CHO, patients would be advised to reduce their intake of Basmati rice and in turn increase their intake of vegetables to meet the recommendations.

In this study, the sources of NSPs were identified, and it was found that fruits were first as a major source of NSPs in patients’ food intakes, followed by whole grain bread. The daily intake of NSP reached 29 ± 10 g/day, which was significantly lower than the recommended amount (13). It is evident that a significant association between a high intake of NSPs and reduced risk of developing type 2 diabetes has been documented (13, 17–18). In fact, the presence of soluble and insoluble NSPs has been shown to reduce FBG and HbA1c and increase insulin sensitivity among patients with type 2 diabetes (27–29). Interestingly, our results demonstrated that a negative correlation between HbA1c and the daily intake of NSPs was observed (*r* = -0.11, *P* < 0.05).

For fruits, the consumption reached approximately 3 servings/day, mostly dates (Rutab and Tamer). This daily intake of fruits met the recommended amount; however, the daily intake of vegetables was not satisfactory.

This study confirmed that the main source of CHOs was Basmati rice followed by breads. Despite the fact that the general consumption of confectionaries and sweetened drinks has recently increased, this study found that the contribution of these items to the total CHOs intake was very low, reaching approximately 3.3% for all sweetened drinks and 2.1% for confectionaries. This trend could be because old individuals (who make up most of our study subjects) have little desire to consume confectionaries and sweetened drinks. It is recommended to minimize the intake of rapidly available CHOs such as refined CHOs and added sugars and increase the intake of slowly available CHOs from vegetables, fruits, whole grains, and legumes (11, 30, 31).

To our knowledge, this study is the first of its kind to document the daily intake of NSPs for Saudi patients with diabetes. In fact, the daily intake of NSPs has not been officially documented for the Saudi population. It is well known that an increased intake of fruits, vegetables, and whole grains has a protective effect against chronic diseases (31).

This study has its natural limitation as a cross-sectional observational study in terms of its ability to demonstrate an association only.

## Conclusion

In this cross-sectional study, it was found that the CHO proportion of food was higher than the recommendation for patients with T2DM, and the major source of their daily intake of CHOs was Basmati rice. Therefore, patients were advised to reduce their intake of Basmati rice Kabsa and increase their consumption of vegetables. A weak negative association between the daily intake of NSPs and HbA1c was observed. Dietary advice should be given to patients to reduce their daily intake of CHOs to maintain their consumption within the recommended daily intake range.
